# Next-Generation Electrically Conductive Polymers: Innovations in Solar and Electrochemical Energy Devices

**DOI:** 10.3390/polym17243331

**Published:** 2025-12-17

**Authors:** Thirukumaran Periyasamy, Shakila Parveen Asrafali, Jaewoong Lee

**Affiliations:** Department of Fiber System Engineering, Yeungnam University, Gyeongsan 38541, Republic of Korea; thirukumaran@ynu.ac.kr (T.P.); shakilaparveen@yu.ac.kr (S.P.A.)

**Keywords:** conjugated polymers, photovoltaic devices, electrochemical storage, sustainable energy, composite electrodes, flexible electronics

## Abstract

The emergence of electrically conductive polymeric materials has revolutionized the landscape of sustainable energy technologies, presenting unprecedented opportunities for advancing both photovoltaic conversion systems and electrochemical energy-storage platforms. These remarkable macromolecular materials exhibit distinctive characteristics including adjustable electronic band structures, exceptional mechanical adaptability, solution-phase processability, and cost-effective manufacturing potential. This extensive review provides an in-depth examination of the fundamental principles governing charge carrier mobility in conjugated polymer systems, explores diverse synthetic methodologies for tailoring molecular architectures, and analyzes their transformative applications across multiple energy technology domains. In photovoltaic technologies, electrically conductive polymers have driven major advancements in organic solar cells and photoelectrochemical systems, significantly improving energy conversion efficiency while reducing manufacturing costs. In electrochemical energy storage, their integration into supercapacitors and rechargeable lithium-based batteries has enhanced charge storage capability, accelerated charge–discharge processes, and extended operational lifespan compared with conventional electrode materials. This comprehensive analysis emphasizes emerging developments in hybrid composite architectures that combine conductive polymers with carbon-based nanomaterials, metal oxides, and other functional components to create next-generation flexible, lightweight, and wearable energy systems. By synthesizing fundamental materials chemistry with device engineering perspectives, this review illuminates the transformative potential of electrically conductive polymers in establishing sustainable, efficient, and resilient energy infrastructures for future technological landscapes.

## 1. Introduction: The Imperative for Advanced Energy Materials

### 1.1. Global Energy Landscape and Sustainability Challenges

The contemporary global society confronts an unprecedented energy crisis characterized by exponentially increasing consumption demands coupled with the progressive depletion of conventional fossil fuel reserves. Current statistical analyses indicate that petroleum-based energy sources continue to dominate worldwide electricity generation and transportation fuel supply chains. However, the environmental consequences of sustained fossil fuel combustion—including atmospheric carbon dioxide accumulation, global temperature elevation, and ecosystem disruption—have catalyzed international initiatives toward renewable energy adoption. Authoritative projections suggest that renewable energy technologies will satisfy approximately one-third of global energy requirements by the mid-21st century, with solar photovoltaic systems positioned as a primary contributor to this transformation [1,2,3,4].

The transition toward sustainable energy infrastructures necessitates revolutionary advances in materials science, particularly in developing cost-effective, scalable, and environmentally benign components for energy conversion and storage devices. Traditional photovoltaic technologies based on crystalline silicon semiconductors, while achieving high conversion efficiencies, suffer from substantial manufacturing costs, rigid mechanical properties, and energy-intensive production processes. Similarly, conventional electrochemical storage systems utilizing metal oxide electrodes and liquid electrolytes face limitations in power density, cycling stability, and environmental safety [5,6,7,8,9].

### 1.2. Emergence of Electrically Conductive Polymers

Electrically conductive polymers represent a paradigmatic breakthrough in materials science, challenging the traditional paradigm that polymeric materials function exclusively as electrical insulators. The discovery that certain conjugated organic macromolecules could exhibit metallic conductivity under appropriate conditions earned Alan Heeger, Alan MacDiarmid, and Hideki Shirakawa the Nobel Prize in Chemistry in 2000, validating the scientific significance of this class of materials. These remarkable substances combine the electronic properties of semiconductors and metals with the mechanical flexibility, lightweight characteristics, and solution processability inherent to polymeric materials. The fundamental electronic structure of conductive polymers derives from extended π-conjugation along the polymer backbone, creating delocalized molecular orbitals that facilitate charge carrier mobility. Through chemical or electrochemical doping processes, these materials can be transformed from semiconducting to metallic states, with electrical conductivities spanning over fifteen orders of magnitude—from insulating (10^−10^ S/cm) to highly conductive (10^5^ S/cm) regimes. This unprecedented tunability, combined with their processability from solution or melt phases, positions electrically conductive polymers as ideal candidates for next-generation energy devices [10,11,12,13].

### 1.3. Scope and Organization of This Review

This comprehensive review systematically examines the multifaceted roles of electrically conductive polymers in advancing thin-film photovoltaic technologies and electrochemical energy-storage systems. The manuscript is organized to provide both fundamental understanding and practical applications perspectives. Initial sections explore the molecular-level origins of electrical conductivity in conjugated polymers, including electronic structure considerations, charge transport mechanisms, and structure-property relationships. Subsequent sections detail synthetic strategies for preparing conductive polymers with tailored properties, encompassing chemical oxidative polymerization, electrochemical deposition, and advanced templating approaches. The review then transitions to device-level applications, beginning with comprehensive analyses of conductive polymer integration into various photovoltaic architectures including dye-sensitized solar cells, organic photovoltaic devices, and hybrid perovskite systems. Particular emphasis is placed on their functions as transparent conducting electrodes, hole transport layers, and electrocatalytic counter electrodes. The energy-storage section examines their implementation in supercapacitors, lithium-ion batteries, and emerging battery chemistries, highlighting performance enhancements achieved through rational materials design. Advanced sections explore cutting-edge developments including multifunctional composite materials combining conductive polymers with carbon nanostructures, metal oxides and other functional additives. The review concludes by identifying persistent challenges, emerging opportunities, and future research directions that will shape the continued evolution of conductive polymer-based energy technologies.

## 2. Fundamental Principles of Electrical Conductivity in Conjugated Polymers

### 2.1. Molecular Origins of Electronic Conductivity

**Figure 1:**
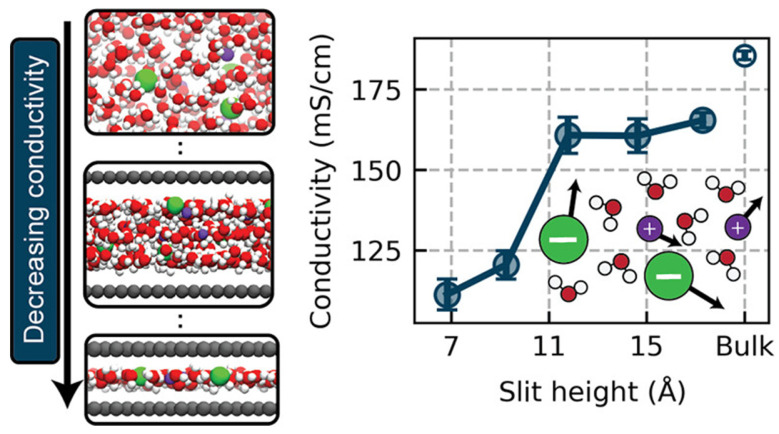
Molecular Origins of Electronic Conductivity mechanism [14].

The exceptional electronic properties of conductive polymers originate from their unique molecular architecture characterized by alternating single and double carbon-carbon bonds along the polymer backbone. This alternating bond structure creates a conjugated π-electron system where p-orbitals on adjacent carbon atoms overlap to form extended molecular orbitals spanning multiple repeat units (Figure 1). In the undoped pristine state, these conjugated polymers typically exhibit semiconducting behavior with bandgaps ranging from 1.5 to 3.5 eV, depending on the specific molecular structure. The electronic structure can be conceptualized using molecular orbital theory, where the highest occupied molecular orbital (HOMO) corresponds to the valence band and the lowest unoccupied molecular orbital (LUMO) represents the conduction band. The energy separation between these frontier orbitals determines the intrinsic electronic properties. For polyacetylene, the archetypal conductive polymer, the conjugated backbone consists of alternating single and double bonds creating a one-dimensional electronic system. However, bond length alternation (Peierls distortion) opens an energy gap, rendering the pristine polymer semiconducting rather than metallic [9,10,11,12].

### 2.2. Doping Mechanisms and Charge Carrier Generation

**Figure 2:**
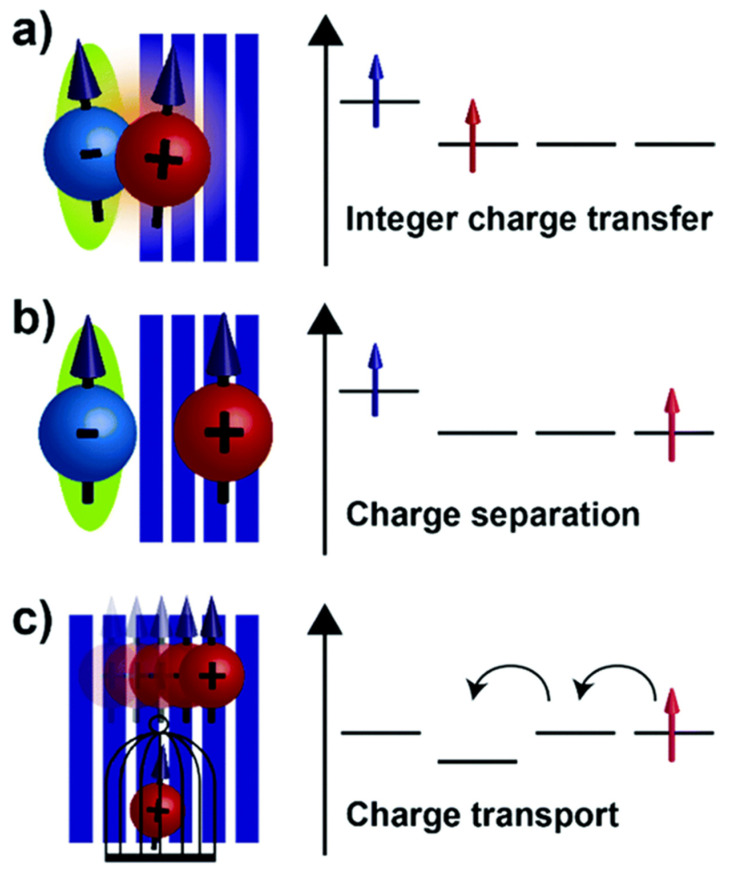
Schematic representation of efficient molecular doping for host material ZnPc (blue rectangles) with p-type dopant F6-TCNNQ (green oval) (**a**) charge transfer state, (**b**) bound charge carriers and (**c**) separated charge carriers [19].

Transforming semiconducting conjugated polymers into conductive materials requires introducing charge carriers through chemical or electrochemical doping. Unlike inorganic semiconductors that use substitutional impurities, polymer doping involves oxidation (p-doping) or reduction (n-doping) of the conjugated backbone, creating mobile carriers such as polarons or bipolarons. P-type doping, the most common method, oxidizes the polymer using electron acceptors like iodine or ferric chloride. Removing an electron forms a radical cation (polaron), and at higher levels, polarons can combine into more stable bipolarons. These species move along and between polymer chains, enabling electrical conductivity, as illustrated in Figure 2 [15,16,17,18]. N-type doping, involving reduction, is less common due to the instability of negatively charged polymers but can be achieved with strong reducing agents for electron-deficient polymers. Conductivity depends on doping level: low concentrations (<1%) yield poor mobility, while moderate levels (5–30%) form continuous conductive paths with near-metallic performance. Excessive doping, however, can disrupt chain order and reduce conductivity [12,13,14,15,16].

### 2.3. Charge Transport Mechanisms

**Figure 3:**
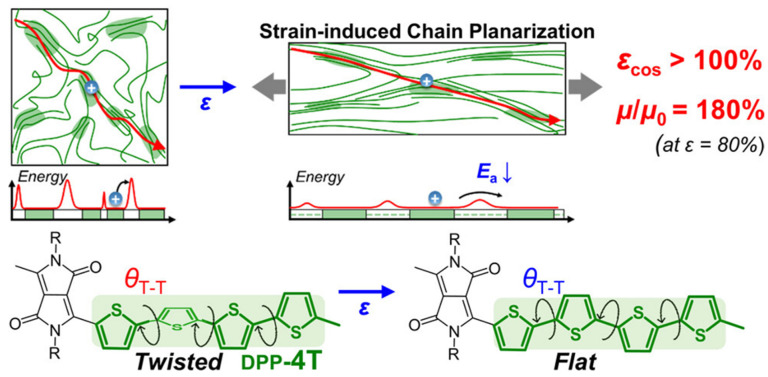
Schematic illustration of Multiscale Analyses of Strain-Enhanced Charge Transport in Conjugated Polymers [24].

Charge transport in conductive polymers involves complex processes operating across multiple length scales. Within individual polymer chains, charge carriers move through delocalized π-orbitals with relatively high mobility. However, macroscopic conductivity requires charge transfer between polymer chains, which occurs through slower hopping or tunneling mechanisms. The overall conductivity therefore depends on both intrachain and interchain transport processes (Figure 3). Several theoretical models have been developed to describe charge transport in conductive polymers. At high temperatures and low doping levels, variable-range hopping dominates, where charge carriers tunnel between localized states with assistance from phonons. This mechanism exhibits characteristic temperature dependence described by the Mott or Efros-Shklovskii variable-range hopping models [20,21,22,23].

Morphological factors critically influence charge transport efficiency. Highly crystalline regions with extended polymer chains and ordered packing facilitate efficient intrachain and interchain transport. Conversely, amorphous domains create energetic barriers and charge carrier traps that impede conductivity. The relative proportions of crystalline and amorphous phases, along with the connectivity between crystalline domains, determine the effective charge carrier mobility.

### 2.4. Structure-Property Relationships

The electronic and transport properties of conductive polymers can be systematically tuned through molecular design strategies that modify the conjugated backbone structure, side chain architecture, and intermolecular packing characteristics. Several structural parameters exert dominant influence on electrical conductivity and other functional properties. Conjugation length, defined as the average number of repeat units over which π-electrons remain delocalized, directly affects the bandgap and charge carrier mobility. Longer conjugation lengths generally correlate with narrower bandgaps and enhanced conductivity. However, structural defects, chain twisting, and conformational disorder limit effective conjugation length in real polymer systems. Rigid, planar backbone structures promote extended conjugation, while flexible or twisted conformations disrupt π-orbital overlap [25,26,27]. Side chain engineering provides a powerful approach for modulating polymer properties without significantly altering the electronic structure of the conjugated backbone. Alkyl side chains enhance solubility and processability but can introduce steric barriers to intermolecular packing. The length, branching, and positioning of side chains must be optimized to balance processability with electronic performance. Functional side chains containing polar groups, ionic moieties, or reactive functionalities enable additional capabilities such as water solubility, self-doping, or crosslinking. Intermolecular organization in the solid state profoundly impacts charge transport efficiency. Face-to-face π-π stacking between conjugated backbones facilitates interchain charge hopping through orbital overlap. The π-π stacking distance, typically 3.4–4.0 Å, critically determines electronic coupling strength. Crystalline polymers with long-range order generally exhibit superior conductivity compared to amorphous materials, although excessive crystallinity can reduce mechanical flexibility [28,29,30,31].

## 3. Synthesis Strategies for Electrically Conductive Polymers

### 3.1. Chemical Oxidative Polymerization

**Figure 4:**
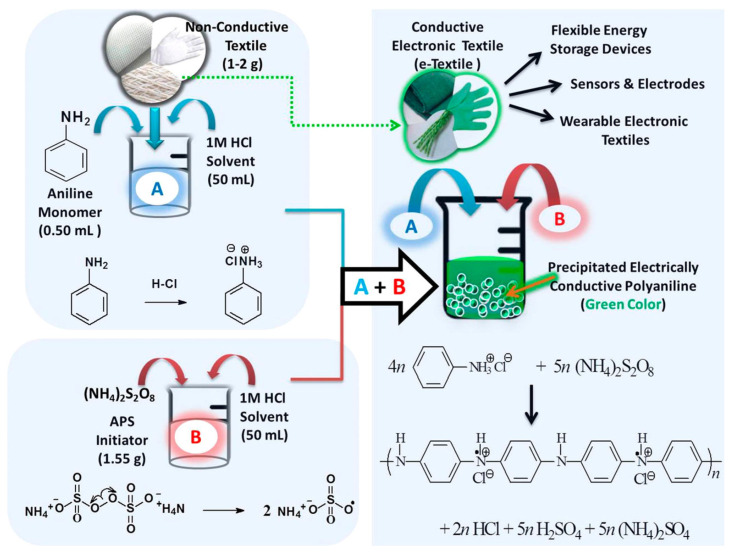
Sketch illustration of the experimental procedure for preparation of conductive polyaniline and conductive polyaniline-coated textiles through in situ chemical oxidative polymerization approach [38].

Chemical oxidative polymerization represents the most widely employed synthetic approach for preparing conductive polymers, offering scalability, simplicity, and compatibility with diverse monomers (Figure 4). This methodology involves oxidation of aromatic or heterocyclic monomers using chemical oxidants, generating radical cations that undergo coupling reactions to form polymer chains. The oxidant simultaneously initiates polymerization and dopes the resulting polymer, yielding conductive materials directly from the reaction mixture [32]. The selection of oxidizing agent critically influences polymerization kinetics, polymer morphology, and final properties. Ferric chloride stands as the most common oxidant for polymerizing thiophene, pyrrole, and aniline derivatives, offering strong oxidizing power and good solubility in organic solvents. Alternative oxidants including ammonium persulfate, hydrogen peroxide, and cerium sulfate provides different reaction kinetics and doping characteristics. The oxidant-to-monomer ratio must be carefully controlled to achieve optimal polymerization while avoiding excessive oxidation that degrades polymer structure.

Reaction conditions including temperature, solvent composition and pH profoundly affect polymer properties. Low-temperature polymerization (0–5 °C) generally produces polymers with longer conjugation lengths and higher conductivity by suppressing side reactions and structural defects. Solvent selection influences polymer morphology through effects on chain solubility and aggregation behavior. Aqueous acidic media favor polyaniline synthesis, while organic solvents such as chloroform or acetonitrile suit polymerization of thiophene derivatives [33,34,35,36,37].

Template-assisted chemical polymerization enables synthesis of conductive polymers with controlled morphologies including nanofibers, nanotubes and core–shell structures. Hard templates such as porous membranes or sacrificial nanostructures provide physical confinement that directs polymer growth. Soft templates including surfactant micelles, polyelectrolytes or biological structures guide polymerization through electrostatic or hydrogen bonding interactions. These templating strategies yield nanostructured conductive polymers with enhanced surface areas and improved electrochemical performance [39].

### 3.2. Electrochemical Polymerization

**Figure 5:**
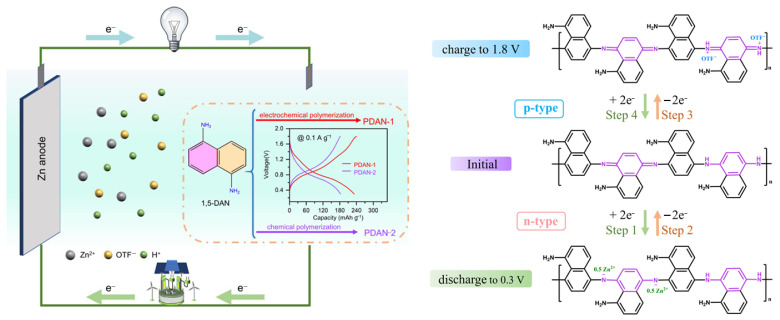
Proposed charge storage mechanism of PDAN-1 [45].

Electrochemical polymerization offers unique advantages for preparing conductive polymer films directly on electrode surfaces, providing excellent adhesion, controlled thickness, and tunable properties. The electro-polymerization mechanism proceeds through several steps: monomer oxidation at the electrode surface generates radical cations, which undergo dimerization through radical coupling or cation-radical coupling (Figure 5). The resulting dimers have lower oxidation potentials than monomers and undergo further oxidation and coupling, propagating polymer chain growth. Simultaneously, the polymer becomes doped with counterions from the electrolyte, establishing electrical conductivity. Electrochemical synthesis parameters provide precise control over film properties. Constant potential (potentiostatic) methods maintain a fixed electrode potential, yielding uniform films with controlled oxidation states. Constant current (galvanostatic) approaches apply fixed current density, enabling faster deposition but results in non-uniform films. Cyclic voltammetry involves repeated potential sweeps, producing multilayer structures with enhanced mechanical stability. Pulse techniques alternating between polymerization and relaxation phases can improve film quality by allowing reorganization between growth cycles [40,41,42,43,44]. The choice of electrolyte system significantly impacts polymer properties. Organic electrolytes based on acetonitrile or propylene carbonate with dissolved salts enable polymerization of monomers with limited aqueous solubility. Aqueous electrolytes offer environmental advantages and facilitate polymerization of water-soluble monomers. Ionic liquids serve as both solvent and electrolyte, providing wide electrochemical windows and unique solvation environments. The electrolyte anion becomes incorporated as the dopant species, influencing conductivity and electrochemical behavior.

### 3.3. Advanced Synthetic Methodologies

Beyond conventional chemical and electrochemical approaches, advanced synthetic strategies have emerged to address specific challenges in conductive polymer preparation. Vapor-phase polymerization enables solvent-free synthesis, where monomer vapors react with oxidant deposited on substrates. This technique produces highly conformal coatings on complex three-dimensional structures and avoids contamination from residual solvents. Oxidative chemical vapor deposition combines monomer and oxidant vapors in a reaction chamber, enabling continuous production of conductive polymer films. Enzymatic polymerization employs oxidoreductase enzymes such as horseradish peroxidase or laccase as biocatalysts for monomer oxidation. This environmentally benign approach operates under mild conditions (ambient temperature, neutral pH, aqueous media) and produces polymers with well-defined structures. Enzymatic synthesis typically yields lower conductivities than chemical methods but offers advantages for biocompatible applications and green chemistry initiatives [46,47,48].

Plasma polymerization utilizes high-energy plasma to activate and polymerize monomers, creating highly crosslinked, adherent coatings with unique properties. While plasma-polymerized materials often exhibit lower conductivities due to structural irregularities, they provide exceptional mechanical durability and chemical stability. Atmospheric pressure plasma systems enable continuous processing without vacuum requirements. Living polymerization techniques including controlled radical polymerization and ring-opening metathesis polymerization have been adapted for synthesizing conjugated polymers with precise molecular weights, narrow polydispersities and controlled architectures. These methods enable preparation of block copolymers combining conductive segments with functional blocks, creating materials with hierarchical self-assembly and multifunctional properties.

The choice of polymerization method depends on the desired application, structural control, and processing requirements. Chemical oxidative polymerization is the most widely used technique for large-scale production due to its simplicity, scalability, and ability to produce conductive polymers directly from reaction mixtures, making it ideal for bulk materials and nanostructures. In contrast, electrochemical polymerization is preferred for forming thin, uniform films directly on conductive substrates, offering precise control over film thickness, doping level, and morphology, which is crucial for applications in sensors, batteries, and supercapacitors. For solvent-free and conformal coatings on complex surfaces, vapor-phase and oxidative chemical vapor deposition methods provide uniformity and purity, while plasma polymerization yields highly adherent and durable coatings with excellent stability. Meanwhile, enzymatic polymerization offers an environmentally friendly route under mild conditions, suitable for biocompatible systems, though it typically results in lower conductivity. Finally, living polymerization techniques enable precise molecular design and formation of block copolymers with tunable electronic and mechanical properties, making them ideal for advanced functional materials.

## 4. Electrically Conductive Polymers in Photovoltaic Technologies

### 4.1. Fundamental Principles of Polymer-Based Photovoltaics

Photovoltaic devices based on organic semiconductors and conductive polymers have emerged as promising alternatives to conventional silicon solar cells, offering advantages including low-cost solution processing, mechanical flexibility, lightweight construction, and tunable optical properties. The operational principles of organic photovoltaics differ fundamentally from inorganic semiconductors due to the molecular nature of light absorption and charge generation. Light absorption in conjugated polymers creates tightly bound electron-hole pairs (excitons) rather than free charge carriers, due to low dielectric constants and strong Coulombic attraction. These excitons must diffuse to interfaces where energetic driving forces facilitate charge separation. The limited exciton diffusion length (typically 10–20 nm) necessitates intimate mixing of electron donor and acceptor materials to ensure efficient charge generation. This requirement has driven development of bulk heterojunction architectures where donor and acceptor phases interpenetrate at nanoscale dimensions [49,50,51]. Following exciton dissociation, separated charges must be transported through their respective phases to collection electrodes while avoiding recombination. Charge transport efficiency depends on the molecular packing, crystallinity, and phase connectivity of the active layer materials. Conductive polymers play multiple critical roles in organic photovoltaic devices: as active layer components for light absorption and charge transport, as interfacial layers for selective charge extraction, and as electrode materials replacing expensive transparent conductors or noble metal catalysts.

### 4.2. Dye-Sensitized Solar Cells: Counter Electrode Applications

**Figure 6:**
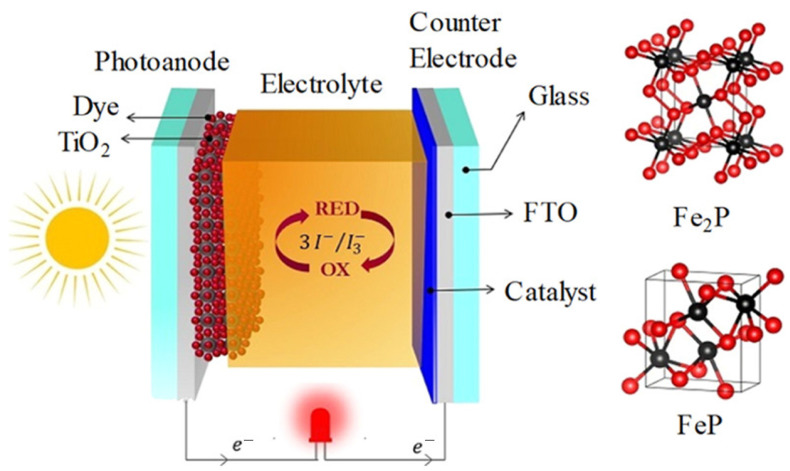
Schematic depiction of working principle of a DSSC [52].

Dye-sensitized solar cells represent a distinct photovoltaic technology where light absorption occurs in molecular dyes adsorbed on mesoporous titanium dioxide, while a liquid or solid electrolyte mediates charge transport, as depicted in Figure 6. Different crystalline phases of iron phosphide were utilized as counter electrodes in dye-sensitized solar cells employing an I^−^/I_3_^−^ electrolyte system. Through solvothermal synthesis with triphenylphosphine as the phosphorus source, Fe_2_P was obtained at 300 °C and FeP at 350 °C. The solar cells incorporating the Fe_2_P counter electrode achieved a power conversion efficiency of 3.96 ± 0.06%, comparable to that of devices using platinum (Pt) electrodes.

Electrically conductive polymers have demonstrated exceptional performance as counter electrode materials in dye-sensitized solar cells, combining high electrocatalytic activity with low cost and facile processing. Polyaniline emerged as an early candidate, exhibiting catalytic activity for iodide/triiodide redox reactions through multiple mechanisms including electrochemical redox processes and chemical interactions with iodine species. The nitrogen-containing backbone provides active sites for iodine coordination, facilitating electron transfer processes. Polypyrrole-based counter electrodes have achieved power conversion efficiencies comparable to platinum references in numerous studies. The porous morphology of electrochemically deposited polypyrrole films provides high surface area for electrocatalytic reactions, while the inherent conductivity ensures efficient electron collection. Optimization of deposition conditions including electrolyte composition, potential and duration enables tailoring of film thickness and porosity for maximum performance. Poly(3,4-ethylenedioxythiophene) or PEDOT represents perhaps the most successful conductive polymer for dye-sensitized solar cell counter electrodes, combining excellent conductivity, transparency and electrocatalytic activity. PEDOT films synthesized by various methods including electrochemical deposition, chemical polymerization and vapor-phase deposition have demonstrated power conversion efficiencies exceeding 8%, rivaling platinum-based devices. The incorporation of poly(styrene sulfonate) as a polymeric dopant (PEDOT:PSS) enhances water dispersibility and film-forming properties, although pure PEDOT generally exhibits superior conductivity [53,54,55,56,57,58].

### 4.3. Composite Counter Electrodes: Synergistic Enhancements

The integration of conductive polymers with carbon-based nanomaterials has yielded dramatic performance enhancements in dye-sensitized solar cell counter electrodes, exploiting synergistic effects between components. Carbon nanotubes, with their exceptional electrical conductivity, high aspect ratios, and large surface areas, provide conductive networks that enhance charge collection and transport. Graphene and reduced graphene oxide offer similar advantages with the additional benefit of two-dimensional morphology that facilitates formation of continuous conductive pathways. Polyaniline-carbon nanotube composites have demonstrated power conversion efficiencies surpassing pure polyaniline by significant margins, with some reports indicating improvements of 30–50%. The carbon nanotubes serve multiple functions: providing conductive pathways that enhance electron collection, increasing surface area for electrocatalytic reactions, and improving mechanical stability of the composite film. The optimal carbon nanotube loading typically ranges from 5 to 20 wt.%, balancing conductivity enhancement against potential aggregation and increased cost. PEDOT-graphene composites represent another highly successful combination, where graphene sheets provide two-dimensional conductive platforms while PEDOT contributes electrocatalytic activity and prevents graphene restacking. These composites have achieved power conversion efficiencies exceeding 8% in multiple studies, with some optimized systems approaching or matching platinum reference devices. The synergistic interaction between PEDOT and graphene creates abundant active sites at interfaces while maintaining high electrical conductivity throughout the film [59,60,61,62].

Metal nanoparticles incorporated into conductive polymer matrices provide additional catalytic activity and conductivity enhancement. Platinum nanoparticles dispersed in PEDOT or polyaniline matrices combine the superior catalytic activity of platinum with the cost advantages and processability of polymers. This approach reduces platinum loading by orders of magnitude compared to conventional platinum-coated electrodes while maintaining comparable performance. Alternative metal nanoparticles including gold, silver and nickel have also demonstrated effectiveness in polymer composite counter electrodes [60,61].

### 4.4. Transparent Conducting Electrodes

**Figure 7:**
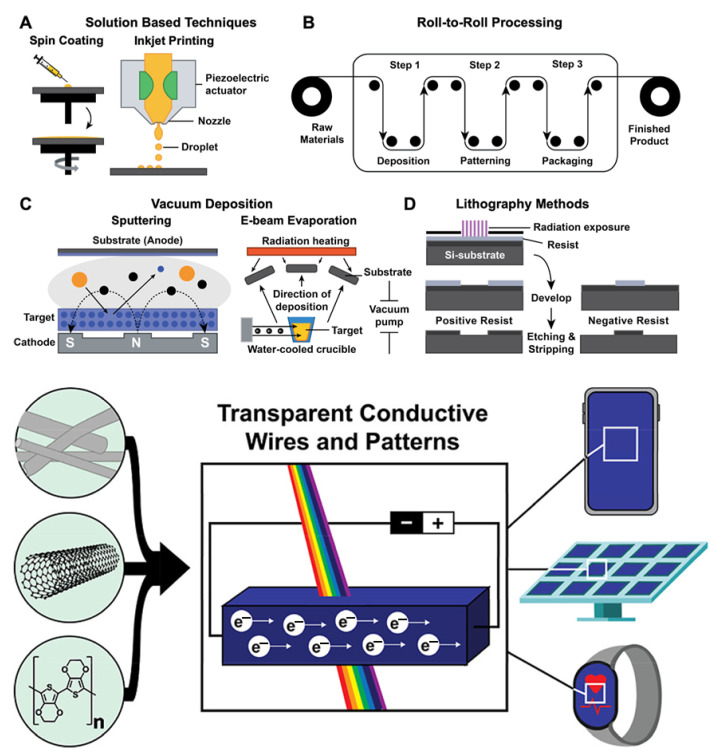
Fabrication methods for transparent conductive patterns and transparent conductive wires and patterns (**A**) solution-based techniques, (**B**) step by step of R2R fabrication, (**C**) vacuum deposition techniques and (**D**) lithography methods [62].

Transparent conducting electrodes serve as essential components in photovoltaic devices, enabling light transmission while collecting photogenerated charges. Indium tin oxide represents the dominant transparent conductor in commercial devices but suffers from brittleness, high cost due to indium scarcity and incompatibility with flexible substrates. Conductive polymers, especially PEDOT-based formulations, have become leading alternatives to traditional transparent conductors due to their mechanical flexibility, solution processability, and tunable optoelectronic properties. Optimized PEDOT:PSS films can achieve sheet resistances below 100 Ω/sq with over 85% optical transmittance in the visible range, rivaling indium tin oxide. Conductivity improvements are achieved through various post-processing strategies, including treatment with polar solvents (e.g., ethylene glycol, dimethyl sulfoxide), incorporation of surfactants or co-solvents during deposition, and thermal or chemical removal of excess insulating PSS (Figure 7). These treatments refine film morphology by enhancing PEDOT chain alignment and promoting phase separation, resulting in improved charge transport and overall performance [32,59,61].

Hybrid transparent electrodes combining PEDOT with metal nanowires (silver or copper) or graphene achieve superior performance through complementary properties. Metal nanowires provide high conductivity but suffer from limited transparency and surface roughness, while PEDOT contributes transparency, smoothness and hole injection properties. The resulting composites exhibit sheet resistances below 20 ohms/square with transmittances exceeding 90%, surpassing indium tin oxide performance while maintaining mechanical flexibility [60,62].

### 4.5. Hole Transport and Interface Engineering

Conductive polymers serve as hole transport layers (HTLs) in organic photovoltaic (OPV) devices, enabling selective extraction of holes from the active layer while blocking electrons. This function is vital for maintaining high open-circuit voltages and fill factors by reducing interfacial charge recombination. Among HTL materials, PEDOT:PSS is widely used due to its suitable work function (~5.0–5.2 eV), excellent film-forming capability, and commercial accessibility. Its energy level alignment with common donor polymers ensures efficient hole transfer and effective charge extraction. However, their intrinsic acidity and hygroscopicity can degrade underlying electrodes and active layers, driving research toward more stable alternative materials. Polyaniline derivatives, polythiophenes with tailored side chains, and crosslinked conductive polymers have demonstrated effectiveness as more stable hole transport layers [63,64,65]. Interface modification through incorporation of functional additives or surface treatments enhances hole transport layer performance. Addition of surfactants modifies surface energy, improving wetting and adhesion of subsequently deposited active layers. Incorporation of metal oxide nanoparticles (tungsten oxide, molybdenum oxide) creates hybrid interface layers with enhanced stability and tunable work functions. Chemical crosslinking of conductive polymer films improves resistance to solvent washing during multilayer device fabrication.

This chapter outlines the essential roles conductive polymers play in solar cell systems, including as transparent electrodes, hole transport layers, and counter-electrodes in DSSCs. Composite strategies combining polymers with carbon nanomaterials or metal nanoparticles significantly enhance catalytic activity, conductivity, and overall device efficiency. These findings underscore the value of polymers in flexible and cost-effective photovoltaic design.

## 5. Electrically Conductive Polymers in Electrochemical Energy Storage

### 5.1. Supercapacitor Applications: Pseudocapacitive Energy Storage

Supercapacitors represent a distinct class of electrochemical energy storage devices bridging the gap between conventional capacitors and batteries, offering high power density, rapid charge–discharge rates, and exceptional cycling stability. Electrically conductive polymers serve as promising electrode materials for supercapacitors, storing charge through fast, reversible redox reactions (pseudocapacitance) in addition to electrostatic double-layer charging. The pseudocapacitive charge storage mechanism in conductive polymers involves rapid, reversible oxidation-reduction reactions accompanied by ion insertion and extraction. During charging, the polymer undergoes oxidation with simultaneous incorporation of anions from the electrolyte to maintain charge neutrality. Discharge reverses this process, reducing the polymer while expelling anions. This redox mechanism enables much higher specific capacitances (100–500 F/g) compared to carbon-based double-layer capacitors (typically 100–200 F/g), although cycling stability may be compromised by structural changes during repeated redox cycling [41,53].

**Figure 8:**
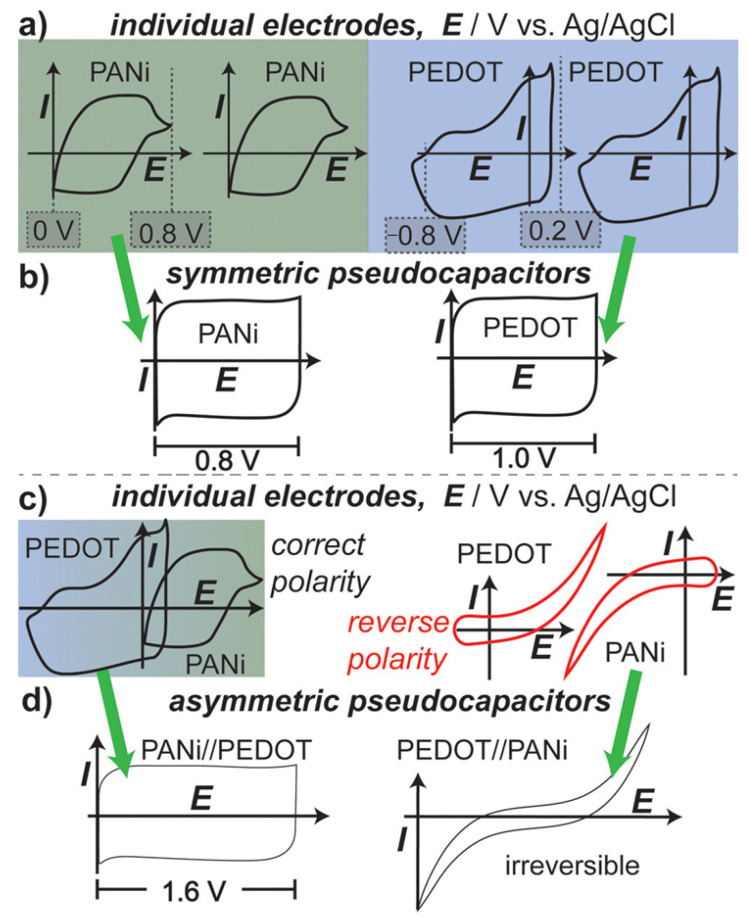
(**a**) 3-Electrode cyclic voltammograms of PANi (**left**) and PEDOT (**right**) in LiClO4 vs. Ag/AgCl. Note: PANi does not exhibit protonation peaks in neutral electrolytes. (**b**) 2-Electrode cyclic voltammograms of electrochemical capacitors using symmetric electrodes of PANi (**left**), and PEDOT (**right**). (**c**) (**Left**) 3-electrode CVs of PANi and PEDOT superimposed. (**Right**) PEDOT and PANi cycled with opposite polarity versus Ag/AgCl show irreversibility and water oxidation/reduction. (**d**) (**Left**) PANi as the positive electrode and PEDOT as the negative electrode in a 2-electrode device shows an extended reversible voltage window. (**right**) PEDOT as the positive electrode and PANi as the negative electrode gives an irreversible, nonideal device [66].

Polyaniline has been extensively investigated as a supercapacitor electrode material due to its high theoretical specific capacitance (approximately 750 F/g), good environmental stability, and multiple accessible oxidation states. The emeraldine form of polyaniline can be reversibly oxidized and reduced, with charge storage occurring through both faradaic redox reactions and proton doping/de-doping processes. Nanostructured polyaniline with high surface area morphologies (nanofibers, nanotubes, porous networks) exhibits enhanced capacitance by providing abundant active sites and facilitating ion transport. Figure 8 compares PANi and PEDOT, showing that PANi exhibits a higher potential stability window, while PEDOT is stable at lower potentials in neutral electrolytes. In symmetric two-electrode configurations (Figure 8b), each material’s operating voltage is restricted by its individual stability range. However, combining PEDOT and PANi in an asymmetric configuration expands the device’s voltage window to 1.6 V. Reversing the polarity drives both materials beyond their stable limits, leading to rapid device failure.

Polypyrrole offers advantages including higher conductivity in the doped state and operation in neutral aqueous electrolytes, avoiding the acidic conditions required for polyaniline. The specific capacitance of polypyrrole typically ranges from 200 to 400 F/g, depending on morphology and measurement conditions. However, polypyrrole suffers from mechanical instability during cycling due to volume changes associated with ion insertion and extraction. Nanostructured morphologies and composite formation with mechanically robust materials help mitigate this limitation. PEDOT-based materials combine high conductivity with good electrochemical stability, making them attractive for supercapacitor applications. The rigid ethylenedioxy substituents on the thiophene rings reduce volume changes during redox cycling, enhancing mechanical stability. PEDOT exhibits specific capacitances of 100–200 F/g, lower than polyaniline but with superior cycling stability, often exceeding 10,000 cycles with minimal capacitance loss [66,67,68].

### 5.2. Composite Electrodes for Enhanced Performance

Combining conductive polymers with carbon nanomaterials, metal oxides, or other functional components produces composite electrodes with superior performance by uniting high pseudocapacitance with excellent conductivity and stability. Polymer–carbon nanotube composites leverage the nanotubes’ electrical conductivity and strength with the polymers’ capacitance, forming 3D conductive networks that enhance charge transport and mechanical durability. Polyaniline–CNT systems have achieved >500 F/g and >90% capacitance retention after 5000 cycles—far exceeding pure polyaniline. Graphene–polymer composites similarly benefit from graphene’s large surface area and flexibility, enabling high interfacial contact and efficient charge transfer. Common synthesis routes include in situ polymerization, electrochemical co-deposition, and layer-by-layer assembly, yielding specific capacitances of 400–600 F/g with excellent stability [33,48,58]. Metal oxide–polymer composites combine the redox activity of oxides (e.g., MnO_2_, RuO_2_, NiO) with the polymers’ conductivity and processability. These hybrids suppress oxide aggregation and improve charge transport. Ternary systems integrating polymers, carbon nanomaterials, and metal oxides have reached capacitances above 800 F/g, nearing theoretical limits while maintaining long-term stability.

### 5.3. Lithium-Ion Battery Applications

**Figure 9:**
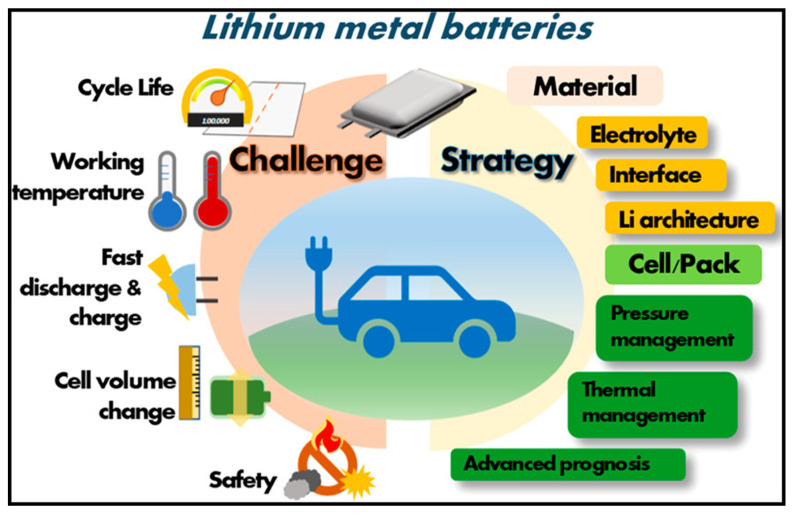
Opportunities and Challenges of High-Energy Lithium Metal Batteries for Electric Vehicle Applications [69].

Lithium-ion batteries represent the dominant rechargeable energy storage technology for portable electronics and electric vehicles, offering high energy density and good cycling performance (Figure 9). Conductive polymers have been explored as electrode materials, binders and electrolyte components in lithium-ion batteries, addressing limitations of conventional materials including limited rate capability, capacity fading, and safety concerns. As anode materials, conductive polymers offer advantages including high theoretical capacity through multiple redox sites, structural flexibility accommodating volume changes, and safe operating potentials avoiding lithium plating. Polyaniline can reversibly store lithium through redox reactions and intercalation processes, with theoretical capacities approaching 300 mAh/g. However, practical capacities remain lower due to incomplete utilization of redox sites and limited electronic conductivity in the reduced state. Nanostructured polyaniline with enhanced surface area and shortened ion diffusion paths exhibits improved capacity and rate performance. Polypyrene-based anodes demonstrate similar characteristics with good cycling stability in certain electrolyte systems. The incorporation of dopants or formation of composites with carbon materials enhances conductivity and capacity. Polythiophene derivatives with tailored side chains have been designed for lithium storage, with some systems achieving reversible capacities exceeding 200 mAh/g over hundreds of cycles [19,26,51].

As cathode materials, conductive polymers face challenges from lower operating voltages compared to transition metal oxides, limiting energy density. However, their structural flexibility, low cost and environmental benignity motivate continued investigation. Conductive polymer cathodes typically operate through redox reactions involving nitrogen or sulfur heteroatoms, with capacities ranging from 100 to 200 mAh/g. Composite cathodes combining conductive polymers with high-voltage materials (lithium iron phosphate, lithium manganese oxide) leverage the conductivity and flexibility of polymers while maintaining high energy density [67,68,69].

### 5.4. Polymer Electrolytes and Separators

Beyond electrode applications, conductive polymers and related polymeric materials serve critical functions as electrolyte hosts and separator membranes in lithium-ion batteries. Solid polymer electrolytes based on polyethylene oxide or polyvinylidene fluoride offer advantages over liquid electrolytes including enhanced safety through elimination of flammable solvents, reduced leakage risk and improved mechanical stability. Gel polymer electrolytes combine liquid electrolyte components absorbed in polymer matrices, achieving ionic conductivities approaching liquid electrolytes while maintaining mechanical integrity. Polyvinylidene fluoride and its copolymers serve as common gel electrolyte hosts due to high dielectric constants, good electrochemical stability and ability to absorb large quantities of liquid electrolyte. The porous structure of electrospun polymer membranes provides high electrolyte uptake and ionic conductivity while maintaining mechanical strength. The incorporation of ionic liquids into polymer electrolytes enhances ionic conductivity, expands electrochemical stability windows, and improves safety through non-volatility and non-flammability. Room-temperature ionic liquids based on imidazolium, pyrrolidinium, or phosphonium cations with various anions provide tunable properties through structural modification. Polymer-ionic liquid gel electrolytes have demonstrated ionic conductivities exceeding 1 mS/cm at room temperature with wide electrochemical windows suitable for high-voltage cathodes [70,71,72,73].

Functionalized conductive polymers with ion-conducting side chains represent an advanced approach to solid polymer electrolytes, where the polymer structure simultaneously provides mechanical integrity and ionic transport pathways. These single-ion conductors eliminate concentration polarization effects present in conventional polymer electrolytes by immobilizing one ionic species while allowing selective transport of the other. Polythiophenes and polyanilines with oligoether or ionic side chains have demonstrated promise in this application, although ionic conductivities remain lower than gel electrolyte systems.

This chapter demonstrates how conductive polymers contribute to supercapacitors and batteries through pseudocapacitive charge storage, redox activity, structural flexibility, and improved rate capability. Composite architectures with carbon materials or metal oxides provide synergetic enhancements, leading to higher capacitance, better stability, and extended cycling life. Conductive polymers also serve as promising anode/cathode hosts and solid/gels in polymer electrolytes.

## 6. Advanced Composite Architectures and Multifunctional Systems

### 6.1. Ternary and Higher-Order Composites

The progression from binary to ternary and higher-order composite systems enables integration of multiple functional components with complementary properties, achieving performance levels unattainable with simpler formulations. These advanced composites typically combine conductive polymers with two or more additional materials selected from carbon nanomaterials, metal oxides, metal nanoparticles and other functional additives. Ternary composites incorporating conductive polymers, graphene and metal oxides have demonstrated exceptional performance in both energy conversion and storage applications. In supercapacitors, polyaniline-graphene-manganese oxide composites achieve specific capacitances exceeding 700 F/g with excellent rate capability and cycling stability. Graphene provides conductive scaffolding and high surface area, manganese oxide contributes additional pseudocapacitance, and polyaniline enhances conductivity while preventing graphene restacking and metal oxide aggregation [74,75,76].

For photovoltaic counter electrodes, ternary composites combining PEDOT, carbon nanotubes, and platinum nanoparticles exploit the catalytic activity of platinum, conductivity of carbon nanotubes and processability of PEDOT. These systems achieve platinum loadings reduced by 90% compared to conventional platinum electrodes while maintaining comparable or superior power conversion efficiencies. The polymer-carbon matrix effectively disperses platinum nanoparticles, maximizing catalytic surface area and preventing sintering.

### 6.2. Hierarchical Nanostructures

**Figure 10:**
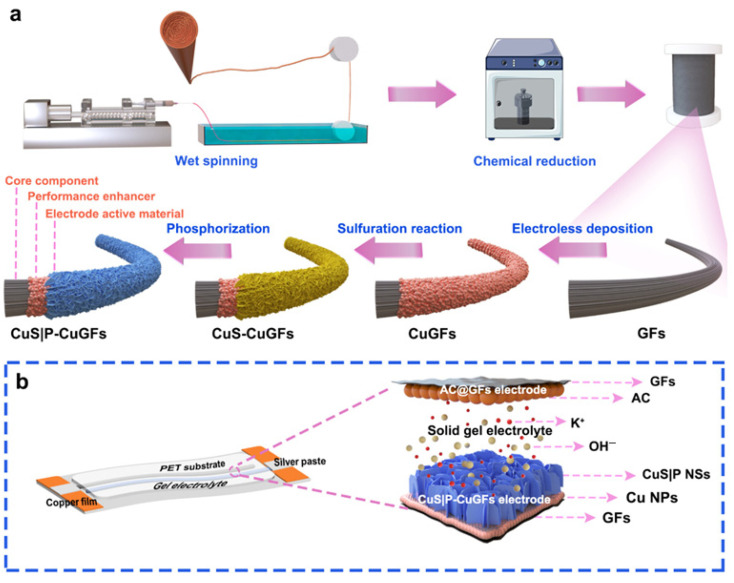
Schematic illustration of (**a**) CuS|P-CuGFs sample fabrication and (**b**) asymmetric FSCs (Fiber based Supercapacitors) [73].

Hierarchical nanostructures featuring multiple length scales of organization enable optimization of competing requirements including surface area, pore accessibility, mechanical stability and charge transport. These architectures typically combine macroscale frameworks providing mechanical support with nanoscale features offering high surface area and short diffusion paths. Three-dimensional porous scaffolds based on carbon foams, metal foams, or textile substrates serve as templates for conductive polymer deposition, creating electrodes with high active material loading and excellent electrolyte accessibility. Electrochemical polymerization on these three-dimensional substrates produces conformal coatings that maintain the open porous structure while incorporating high capacitance materials. The resulting electrodes exhibit areal capacitances exceeding 1 F/cm^2^ while maintaining good rate capability. Core–shell nanostructures with conductive polymer shells on carbon nanotube or metal oxide cores combine the mechanical and electrical properties of the core with the pseudocapacitance of the polymer shell (Figure 10). The core provides structural support preventing polymer swelling and contraction during redox cycling, while the thin polymer shell ensures short ion diffusion paths and complete utilization of active material. Consequently, the CuS|P-CuGF (copper phosphosulfide nanosheets–Cu-coated graphene fiber) fiber electrodes achieved excellent electrical conductivity (697.8 S cm^−1^), strong mechanical durability (285.0 ± 8.4 MPa), and outstanding electrochemical properties. When integrated into an asymmetric supercapacitor, the device exhibited an areal energy density of 15.3 μWh cm^−2^ at a power density of 1.1 mW cm^−2^, maintaining 94.3% capacitance retention after 500 bending cycles (R = 10 mm), demonstrating excellent flexibility and stability. These architectures achieve specific capacitances approaching theoretical values with cycling stabilities exceeding 10,000 cycles [68,69,70,71,72,73].

### 6.3. Flexible and Stretchable Energy Devices

**Figure 11:**
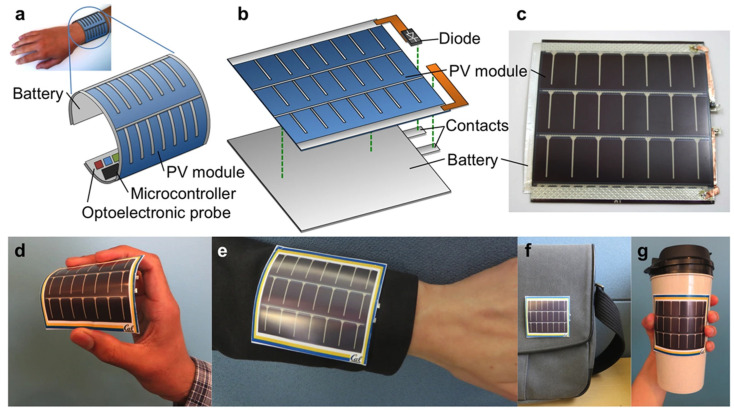
(**a**) Illustration of activity-tracking wristband concept containing flexible battery, PV energy harvesting module, and pulse oximeter components. (**b**) Diagram and (**c**) photograph of a flexible energy harvesting and storage system comprising PV module, battery, and surface-mount Schottky diode, showing the components and attachment points. The diode is included to prevent discharge of the battery into the PV module in low-light conditions. (**d**–**g**) Photographs of the device being flexed in the hand (**d**) and on various f lexible and curved surfaces: jacket sleeve (**e**), bag (**f**), and travel mug (**g**) [79].

The mechanical flexibility and solution processability of conductive polymers enable fabrication of flexible and stretchable energy devices for wearable electronics, implantable sensors and conformable power sources. These applications require materials that maintain functionality under mechanical deformation including bending, twisting and stretching. Flexible supercapacitors based on conductive polymer electrodes deposited on flexible substrates (polymer films, textiles, paper) demonstrate stable performance under repeated bending cycles. The battery, depicted in Figure 11, features printed graphite anode and lithium cobalt oxide cathode layers on thin, flexible current collectors. It delivers an energy density of 6.98 mWh cm^−2^ and maintains 90% capacity at a 3C discharge rate, with nearly 99% retention after 100 charge–discharge cycles and 600 mechanical bending cycles, highlighting its excellent electrochemical and mechanical stability. The intrinsic flexibility of polymer chains accommodates substrate deformation without cracking or delamination. Device architectures including fiber-shaped, planar and three-dimensional configurations have been developed for different application requirements. Fiber supercapacitors incorporating conductive polymer electrodes on carbon fiber or metal wire substrates achieve volumetric capacitances exceeding 10 F/cm^3^ while maintaining flexibility for textile integration. Stretchable energy devices require materials and architectures that accommodate large strain deformations (greater than 50% elongation) while maintaining electrical and electrochemical functionality. Strategies for achieving stretchability include intrinsically stretchable conductive polymers with flexible backbones and side chains, composite materials combining rigid conductive elements with elastomeric matrices, and structural designs featuring wavy, serpentine, or island-bridge configurations that convert stretching into bending or unfolding motions. Intrinsically stretchable conductive polymers based on polythiophenes with flexible alkyl side chains or elastomeric backbones maintain conductivity under strains exceeding 100%. These materials enable fully stretchable electrodes and interconnects for energy devices. Composite approaches embedding conductive polymer nanostructures in elastomeric matrices (polydimethylsiloxane, polyurethane) create percolating conductive networks that maintain connectivity during stretching. These composites achieve conductivities of 10–100 S/cm with stretchability exceeding 200% [74,75,76,77,78].

### 6.4. Self-Healing and Adaptive Materials

Self-healing conductive polymers capable of autonomously repairing mechanical damage represent an emerging frontier for enhancing device reliability and longevity. These materials incorporate dynamic chemical bonds or physical interactions that can reversibly break and reform, enabling recovery of mechanical and electrical properties after damage. Conductive polymers with dynamic covalent bonds including Diels-Alder adducts, disulfide linkages, or imine bonds exhibit self-healing behavior through thermally activated bond exchange reactions. Upon damage, heating above a threshold temperature activates bond breaking and reformation, allowing polymer chains to reconnect across damaged interfaces. These materials demonstrate recovery of mechanical strength exceeding 90% after healing cycles, with conductivity recovery depending on the extent of conjugation disruption. Supramolecular conductive polymers utilizing non-covalent interactions (hydrogen bonding, metal coordination, π-π stacking) achieve self-healing at ambient temperature through dynamic association and dissociation of molecular recognition motifs. These materials flow and reform under stress, enabling healing without external stimuli. The trade-off between self-healing capability and mechanical strength requires careful molecular design to balance interaction strength with dynamic reversibility [80,81,82,83].

This chapter illustrates how ternary composites, hierarchical nanostructures, flexible substrates, and self-healing materials enable multifunctional energy devices. The combination of conductive polymers with carbon frameworks, metal oxides, elastomers, or dynamic bonds results in systems that are mechanically resilient, high-performing, and suitable for wearable or deformable electronics.

## 7. Characterization Techniques and Performance Metrics

### 7.1. Structural and Morphological Characterization

**Figure 12:**
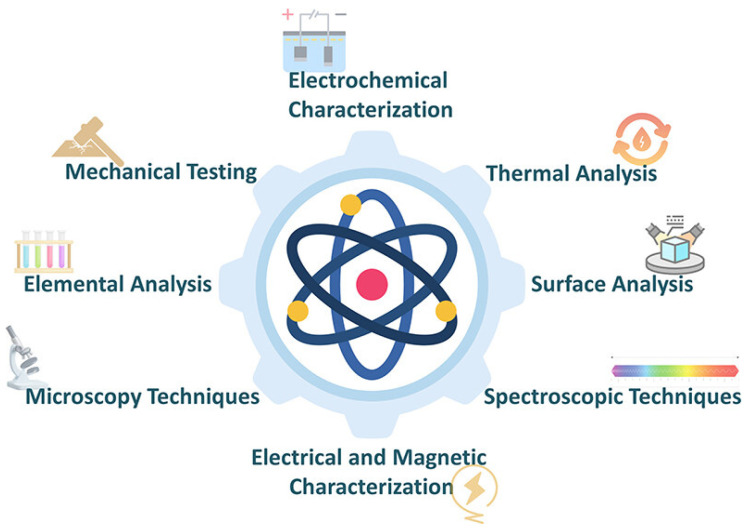
Mastering Material Insights: Advanced Characterization Techniques [84].

Comprehensive characterization of conductive polymer materials requires multiple complementary techniques to elucidate structure across length scales from molecular to macroscopic (Figure 12). Spectroscopic methods provide information about chemical structure, electronic states and molecular interactions, while microscopy techniques reveal morphology and microstructure. Ultraviolet–visible–near-infrared spectroscopy probes the electronic structure of conductive polymers through optical absorption arising from π-π* transitions and polaron/bi-polaron absorption bands. The position and intensity of these absorption features indicate conjugation length, doping level and electronic structure. Doped conductive polymers exhibit characteristic absorption in the near-infrared region associated with charge carriers, providing a non-destructive method for assessing doping efficiency. Fourier transform infrared spectroscopy identifies functional groups and monitors chemical changes during synthesis, doping or device operation. Characteristic vibrations of conjugated backbones, dopant species and side chains provide fingerprints for molecular structure determination. In situ infrared spectroscopy during electrochemical cycling reveals dynamic changes in oxidation state and dopant incorporation. Raman spectroscopy offers high sensitivity to conjugated backbone structure with spatial resolution enabling mapping of composition and doping in composite materials. The intensity ratio of characteristic Raman bands correlates with conjugation length and crystallinity. Resonance Raman enhancement when excitation wavelength matches electronic transitions provides selective amplification of signals from specific molecular species. X-ray diffraction characterizes crystalline structure, including lattice parameters, crystallite size and preferred orientation [28,29,30,31,32,33]. Conductive polymers typically exhibit limited crystallinity with broad diffraction peaks reflecting small crystallite dimensions and structural disorder. The π-π stacking distance between conjugated backbones, critical for charge transport, can be determined from diffraction patterns. Grazing incidence X-ray diffraction provides surface-sensitive structural information for thin films [24]. Electron microscopy techniques including scanning electron microscopy and transmission electron microscopy reveal morphology, particle size and composite structure at nanometer resolution. Scanning electron microscopy characterizes surface topography and film thickness, while transmission electron microscopy enables visualization of internal structure and interfaces in composite materials. Energy-dispersive X-ray spectroscopy coupled with electron microscopy provides elemental composition and distribution information.

### 7.2. Electrical and Electrochemical Characterization

Electrical conductivity measurements quantify the charge transport properties fundamental to conductive polymer applications. Four-point probe techniques eliminate contact resistance effects, providing accurate conductivity values for films and pellets. Temperature-dependent conductivity measurements reveal charge transport mechanisms, with different temperature dependences indicating hopping, tunneling, or metallic transport regimes. Electrochemical characterization techniques assess the redox behavior, charge storage capacity and stability of conductive polymers in energy device applications. Cyclic voltammetry maps the oxidation and reduction potentials, providing information about accessible redox states and electrochemical reversibility. The shape and position of voltammetric peaks indicate the nature of charge storage processes (capacitive versus battery-like) and the kinetics of redox reactions [85].

Galvanostatic charge–discharge measurements determine specific capacity, energy density, power density and cycling stability under conditions mimicking device operation. The shape of charge–discharge curves distinguishes between capacitive behavior (linear voltage–time relationship) and battery-like behavior (voltage plateaus). Coulombic efficiency, defined as the ratio of discharge to charge capacity, indicates the reversibility of charge storage processes. Electrochemical impedance spectroscopy probes the frequency-dependent response of electrochemical systems, separating contributions from charge transfer resistance, ionic resistance and capacitive elements. Nyquist plots displaying imaginary versus real impedance components reveal kinetic and transport limitations. The high-frequency semicircle diameter correlates with charge transfer resistance, while the low-frequency region indicates capacitive or diffusion-controlled behavior [39,40,41,42,43,44,45].

### 7.3. Photovoltaic Device Characterization

Current-voltage characterization under simulated solar illumination provides the fundamental performance metrics for photovoltaic devices: short-circuit current density, open-circuit voltage, fill factor and power conversion efficiency. The short-circuit current density reflects the photocurrent generation and collection efficiency, depending on light absorption, exciton dissociation and charge transport. Open-circuit voltage indicates the maximum voltage achievable, determined by the energy level offset between donor and acceptor materials and recombination losses. Fill factor, defined as the ratio of maximum power point to the product of short-circuit current and open-circuit voltage, quantifies the quality of charge extraction and the influence of series and shunt resistances. High fill factors (above 70%) indicate efficient charge collection with minimal resistive losses. Power conversion efficiency, the ratio of electrical power output to incident solar power, represents the overall device performance metric [52,53,54,55,56,57,58].

External quantum efficiency spectroscopy measures the wavelength-dependent photocurrent generation efficiency, revealing the contribution of different device components to overall current generation. Comparison of external quantum efficiency spectra with absorption spectra identifies loss mechanisms including incomplete light absorption, inefficient exciton dissociation, or poor charge collection at specific wavelengths. Stability testing under continuous illumination, elevated temperature, or ambient atmosphere exposure assesses device longevity and degradation mechanisms. Tracking performance parameters over hundreds or thousands of hours reveals failure modes including active layer degradation, electrode corrosion, or encapsulation failure. Accelerated aging protocols employing elevated temperature and humidity provide predictions of operational lifetime [86,87]. A comparative summary of polymer types, including their performance benchmarks, advantages and limitations are included in Table 1.

## 8. Challenges, Opportunities and Future Perspectives

### 8.1. Stability and Degradation Mechanisms

Despite remarkable progress in performance, long-term stability remains a critical challenge limiting commercial adoption of conductive polymer-based energy devices. Multiple degradation mechanisms contribute to performance decline including chemical oxidation, structural reorganization, dopant loss and interfacial degradation. Chemical oxidation by atmospheric oxygen and moisture represents a primary degradation pathway, particularly for polymers in reduced states or with low ionization potentials. Oxidation reactions disrupt conjugation, create defects and reduce conductivity. Strategies to enhance oxidative stability include molecular design incorporating electron-withdrawing substituents that lower HOMO energy levels, encapsulation to exclude oxygen and moisture, and addition of antioxidants or radical scavengers. Mechanical degradation during electrochemical cycling arises from volume changes accompanying redox reactions and ion insertion-extraction. Repeated swelling and contraction can cause delamination, cracking and loss of electrical connectivity. Specifically, Polyaniline and Polypyrrole exhibit poor long-term cycling stability, a critical drawback for energy storage applications. This degradation is primarily caused by volumetric changes that occur as ions are repeatedly inserted and removed during charging and discharging (the doping/de-doping process). These mechanical stresses lead to the pulverization and cracking of the active film, resulting in a progressive loss of electrical conductivity and capacitance. Furthermore, the widely used PEDOT:PSS formulation suffers from a different set of material challenges: it is highly acidic and moisture-sensitive. The material’s acidity can cause undesirable side reactions and degradation of underlying layers in complex optoelectronic devices, while its hygroscopicity negatively impacts long-term performance and reliability under ambient conditions [9,10]. Composite materials with mechanically robust scaffolds, crosslinking to improve mechanical integrity, and nanostructured morphologies with strain accommodation capability mitigate mechanical degradation. Dopant loss through diffusion or chemical reactions reduces conductivity and alters electronic properties. The electrical function of conductive polymers relies entirely on the presence of mobile dopant ions, yet the instability and migration of these dopants pose a major obstacle to device longevity. In an operating device, an applied electric field creates a potential gradient across the polymer film, which acts as a driving force for the movement of the charge-compensating counterions. This dopant migration leads to the formation of a highly inhomogeneous dopant distribution within the material over time. This resulting non-uniformity causes the film’s electrical conductance to change spontaneously and degrade, undermining the operational stability of the final electronic or electrochemical device, such as actuators and organic transistors, where stable charge density is paramount. Ionic dopants can migrate under electric fields or concentration gradients, particularly at elevated temperatures. Covalent attachment of dopant species to polymer backbones (self-doping) prevents migration, although this may compromise other properties. Polymeric dopants with large molecular size exhibit reduced mobility compared to small-molecule dopants [11,12].

### 8.2. Scalable Manufacturing and Processing

Transitioning conductive polymer technologies from laboratory demonstrations to commercial production requires development of scalable, reproducible and cost-effective manufacturing processes. Solution processing methods including spin coating, blade coating, slot-die coating and inkjet printing enable large-area deposition on flexible substrates using roll-to-roll manufacturing. Ink formulation represents a critical challenge, requiring optimization of polymer concentration, solvent selection, viscosity and surface tension for specific deposition methods. Conductive polymer inks must maintain stability during storage, deposit uniformly without defects, and form continuous films with desired properties after drying. Additives including surfactants, rheology modifiers and adhesion promoters enable control over ink properties and film formation [38,39,40,41,42,43,44,45].

While traditional chemical polymerization methods are often scalable, the advanced synthesis techniques required to achieve high-performance polymer films have significant scalability limitations. Electrochemical polymerization provides excellent control over film thickness and morphology with high substrate adhesion, but its reliance on electrode surfaces inherently restricts it to batch processing and small areas, making it unsuitable for continuous, large-area manufacturing required for commercial displays. Similarly, Vapor-Phase Polymerization (VPP) is a crucial solvent-free technique for depositing highly conformal films on sensitive substrates. However, VPP is technically complex due to the difficulty of tuning numerous interconnected process parameters (monomer, oxidant, and temperature), which hinders the achievement of the uniform film thickness and performance necessary for cost-effective, high-throughput industrial scale-up. Quality control and process monitoring become increasingly important at commercial scales. In-line characterization techniques including optical spectroscopy, conductivity mapping and thickness measurement enable real-time process optimization and defect detection. Statistical process control methods ensure batch-to-batch reproducibility and identify sources of variation.

### 8.3. Environmental and Sustainability Considerations

The environmental impact of materials and processes across the entire lifecycle—from raw material extraction through manufacturing, use, and end-of-life disposal—must be considered for truly sustainable energy technologies. Conductive polymers offer potential advantages in sustainability compared to conventional materials, but challenges remain. Green chemistry approaches to conductive polymer synthesis minimize environmental impact through use of benign solvents (water, bio-derived solvents), renewable feedstocks, and elimination of toxic reagents. Enzymatic polymerization and bio-derived monomers represent promising directions. However, many high-performance conductive polymers currently require organic solvents or toxic oxidants for synthesis. For instance, Ferric chloride is a widely used oxidant for PPy, resulting in iron-containing wastewater that requires complex remediation. Furthermore, the monomer used for PANI synthesis, Aniline, is itself a known environmental toxin derived from non-renewable petroleum feedstocks. These traditional methods violate Green Chemistry principles by introducing hazardous materials and operating under harsh, energy-intensive conditions (e.g., highly acidic media). While research is advancing towards enzymatic polymerization using “greener” oxidants like hydrogen peroxide and catalysts like Horseradish Peroxidase, these alternatives currently face hurdles in achieving the high conductivity and yield necessary for industrial scale-up. Recycling and end-of-life management of conductive polymer-based devices require development of separation and recovery processes. The complex composite structures in high-performance devices complicate recycling efforts. The incorporation of nanofillers (e.g., carbon nanotubes, graphene, metal nanoparticles) into the polymer matrix results in an intrinsically inhomogeneous material system. Conventional recycling methods, such as mechanical recycling (shredding and remelting), typically degrade the material’s properties and destroy the critical conductive network, leading to downcycling rather than reuse. The chemical separation of the nano-component from the polymer matrix is often energy-intensive and requires corrosive solvents (e.g., in solvolysis). Moreover, the small size of the nanoparticles raises concerns about their potential release into the environment during disposal or incomplete recycling processes, where they may interact with biological systems [52,53,54,55,56]. Design for disassembly, using reversible adhesives and easily separable components, facilitates material recovery. Chemical recycling processes that depolymerize conductive polymers to recover monomers offer potential for closed-loop manufacturing. Molecular dopants, critical for achieving high electrical conductivity, pose a distinct environmental risk, particularly those that are highly effective p-type dopants. The strong oxidizing agent, F6-TCNNQ, exemplifies this concern. Its high electron affinity is achieved through extensive fluorine substitution (perfluorination). This structural feature links it to the class of per- and polyfluoroalkyl substances (PFAS), or “forever chemicals,” which are known for their extreme environmental persistence and potential for bioaccumulation. Although F6-TCNNQ enhances device performance, its presence means that, upon the inevitable disposal of the electronic device, the dopant (or its degradation products) is a persistent pollutant that poses a long-term, non-biodegradable risk to ecosystems and human health. Life cycle assessment provides quantitative evaluation of environmental impacts including energy consumption, greenhouse gas emissions, water usage, and toxicity across all lifecycle stages. Comparative assessments of conductive polymer technologies versus conventional alternatives reveal trade-offs and identify opportunities for improvement. Transparent reporting of lifecycle impact enables informed decision-making and drives development toward more sustainable solutions [57,58].

### 8.4. Integration with Emerging Technologies

The convergence of conductive polymers with other emerging technologies creates opportunities for transformative applications beyond traditional energy conversion and storage. Integration with internet-of-things devices, artificial intelligence, and advanced manufacturing techniques enables new functionalities and applications. Smart energy systems incorporating sensors, communication capabilities, and adaptive control require flexible, distributed power sources that conductive polymer-based devices can provide. Printed batteries and supercapacitors integrated with flexible sensors and wireless communication modules enable self-powered sensor networks for environmental monitoring, structural health monitoring and biomedical applications [79].

Artificial intelligence and machine learning approaches accelerate materials discovery and optimization by identifying structure-property relationships from large datasets. High-throughput computational screening of candidate polymer structures, combined with automated experimental synthesis and characterization, enables rapid exploration of chemical space. Machine learning models trained on existing data predict properties of novel materials, guiding experimental efforts toward promising candidates.

Additive manufacturing techniques including three-dimensional printing enable fabrication of complex device geometries and customized designs impossible with conventional manufacturing. Direct ink writing of conductive polymer composites creates three-dimensional electrode architectures with controlled porosity and tortuosity. Multi-material printing enables integration of electrodes, electrolytes, and current collectors in single manufacturing steps. Bio-integrated energy devices leveraging the biocompatibility and mechanical compliance of conductive polymers enable new applications in implantable medical devices, bioelectronics and human–machine interfaces. Conductive polymers with ionic-electronic coupling facilitate communication with biological systems through both electronic and ionic signals. Biodegradable conductive polymers based on natural polymers or designed with cleavable linkages enable transient devices that safely dissolve after their functional lifetime [74,75,76].

### 8.5. Fundamental Research Directions

Despite extensive investigation, fundamental aspects of conductive polymer physics and chemistry remain incompletely understood, presenting opportunities for discovery-driven research that could enable breakthrough advances. The detailed mechanisms of charge transport in disordered conjugated polymers, particularly the interplay between intrachain and interchain transport, require further elucidation. Advanced spectroscopic techniques with femtosecond time resolution probe charge carrier dynamics and relaxation processes. Single-molecule and single-chain measurements reveal heterogeneity and provide insights into intrinsic properties without ensemble averaging. Structure-property relationships in conductive polymers involve complex coupling between molecular structure, solid-state organization and functional properties. Systematic studies varying molecular parameters (conjugation length, side chain structure, backbone planarity) while controlling processing conditions enable isolation of specific effects. Computational modeling at multiple scales from quantum chemistry to device-level simulations complements experimental investigations [9,19,24,38,45].

Interfacial phenomena at heterojunctions between conductive polymers and other materials critically determine device performance but remain challenging to characterize and control. Advanced microscopy and spectroscopy techniques with nanometer spatial resolution map composition, electronic structure, and charge transfer dynamics at interfaces. Molecular-level control over interface formation through self-assembly or directed assembly enables optimization of interfacial properties. Novel polymer architectures including conjugated polyelectrolytes, conjugated ladder polymers, and conjugated metal–organic frameworks expand the structural diversity beyond conventional linear conjugated polymers. These materials exhibit unique properties arising from their distinct architectures, potentially enabling new applications or enhanced performance in existing applications [52,62].

Recent advances show that the conductivity of intrinsically low-conductive polymers can be significantly improved through complexation with polyelectrolytes, dopant acids, or ionic species. For example, the well-known PEDOT:PSS system demonstrates how strong Coulombic interactions between PEDOT chains and the sulfonate groups of PSS induce improved chain ordering, phase separation, and enhanced charge carrier mobility. Similar complexation strategies—such as incorporating protonic acids, polymeric sulfonates, or ionic liquid dopants—can promote structural rearrangement, increase doping efficiency, and create continuous percolation pathways for charge transport. Importantly, these complexes not only boost conductivity but also enhance environmental and electrochemical stability by suppressing dopant loss, reducing morphological degradation, and improving moisture tolerance [5,66,71]. Applying such molecular complexation principles to emerging conductive polymers therefore offers a powerful route to simultaneously optimize conductivity and long-term durability for photovoltaic and electrochemical energy-storage applications.

## 9. Conclusions and Outlook

Electrically conductive polymers have emerged as transformative materials for sustainable energy technologies, enabling advances in both photovoltaic conversion systems and electrochemical energy storage devices. Their unique combination of tunable electronic properties, mechanical flexibility, solution processability, and low-cost manufacturing potential positions them as key enablers of next-generation energy systems. In photovoltaic applications, conductive polymers have demonstrated effectiveness as transparent conducting electrodes, hole transport layers, and electrocatalytic counter electrodes, contributing to efficiency enhancements of up to 20% and cost reductions exceeding 30% in various device architectures [59,60,61,62]. Composite materials combining conductive polymers with carbon nanomaterials, metal nanoparticles, or metal oxides achieve synergistic performance improvements, with some systems matching or exceeding conventional platinum-based electrodes while dramatically reducing precious metal consumption.

For energy storage applications, conductive polymers serve as high-capacity pseudocapacitive materials in supercapacitors and as electrode materials, binders, and electrolyte hosts in batteries. Integration into composite architectures has yielded specific capacity improvements of 50%, rate capability enhancements of 40%, and cycling stability extensions of 60% compared to conventional materials [74,75,76,77,78]. These performance gains translate to practical devices with enhanced energy density, power capability and operational lifetime.

Despite remarkable progress, significant challenges remain before widespread commercial adoption of conductive polymer-based energy technologies. Stability under operational conditions, particularly long-term cycling stability and environmental stability, requires continued improvement through molecular design, composite engineering and protective encapsulation strategies. Scalable manufacturing processes must be developed and optimized to enable cost-effective production while maintaining performance and reproducibility. Environmental sustainability across the entire lifecycle, from synthesis through end-of-life disposal, demands attention to green chemistry principles, renewable feedstocks and recycling strategies. The future trajectory of conductive polymer energy technologies will likely emphasize multifunctional systems combining energy conversion, storage, and utilization in integrated platforms. Flexible and stretchable devices enabling wearable electronics, conformable sensors, and implantable medical devices represent particularly promising application domains leveraging the unique mechanical properties of polymers. Integration with emerging technologies including artificial intelligence for accelerated materials discovery, additive manufacturing for complex device architectures, and bioelectronics for human–machine interfaces will expand the application space and impact of conductive polymers. Fundamental research continuing to elucidate charge transport mechanisms, structure-property relationships, and interfacial phenomena will provide the knowledge base for rational materials design and performance optimization. Novel polymer architectures expanding beyond conventional structures may unlock new properties and capabilities. The convergence of advances in synthesis, characterization, theory, and device engineering promises continued progress toward realizing the full potential of electrically conductive polymers in enabling sustainable, efficient, and resilient energy systems for future technological landscapes. The field of electrically conductive polymers for energy applications has matured significantly over the past decades, transitioning from fundamental discovery to practical device demonstrations and early commercialization. However, substantial opportunities remain for innovation and improvement. By addressing persistent challenges while exploring new frontiers, the research community can accelerate the translation of conductive polymer technologies from laboratory curiosities to ubiquitous components of global energy infrastructure, contributing meaningfully to the urgent need for sustainable energy solutions.

## Figures and Tables

**Table 1:** Comparison of Properties of Conductive Polymers in Energy Applications.

Category/Material System	Key Structural Features	Charge Transport/Conductivity	Functional Advantages	Main Limitations	Typical Applications	Ref
Small-Molecule OSCs (e.g., ZnPc:F6-TCNNQ)	Integer charge transfer; polaron formation; dopant–host interactions	CT-limited conductivity; low activation energy (~5–233 meV)	High doping efficiency; clear spin signatures	Dopant aggregation; Coulomb binding	Organic electronics, sensors	[19]
Rigid Conjugated Polymers (e.g., DPP-DTT)	High backbone rigidity; strong π–π stacking	High baseline mobility, limited stretch response	Strong crystallinity; efficient transport	Poor stretchability	OFETs, thin-film electronics	[24]
Polyaniline/Conducting Polymers	π-conjugated backbone; acid doping	High conductivity when protonated	Low-cost; textile integration	Exothermic polymerization; variable uniformity	Wearables, pseudocapacitors	[38]
Electropolymerized Polymers (e.g., PDAN-1)	Controlled growth; extended conjugation	High specific capacity (243 mAh g^−1^)	Superior stability and conductivity	Requires electrochemical setup	Aqueous batteries, hybrid capacitors	[45]
Metal Phosphides (Fe_2_P, FeP)	High catalytic active-site density	Good I_3_^−^ reduction kinetics	Pt-free, low-cost catalysts	Performance varies with stoichiometry	DSSC counter electrodes	[52]
Hydrogels—Nanocomposite	Polymer + CNT/graphene/clay	High conductivity (with fillers)	Multifunctional, tunable	Dispersion challenges	Flexible electronics, biosensors	[62]
Hydrogels—Conductive Polymer	PEDOT/PPy/PANi integrated	Very high conductivity	Great for bioelectrodes	Brittle without soft matrix	Neural interfaces, E-skins	[62,66]
Transparent Conductors (ITO, FTO, AZO)	Metal oxide networks	Rs as low as <10 Ω/sq	High transparency	Vacuum deposition needed	PVs, displays, smart windows	[73]
Metal Nanowire Networks (Ag NWs)	Percolating metal networks	High conductivity	Printable, transparent	Junction resistance, corrosion	Wearables, touch sensors	[73]
Carbon Nanomaterials (CNT, graphene)	1D/2D sp^2^ networks	Moderate conductivity	Ultra-thin, stretchable	Higher sheet resistance	Soft electronics	[62]
Flexible Li-ion Batteries	Graphite/LCO thin layers	High areal capacity	Thin, flexible	Limited by encapsulation	Wearable medical devices	[79]
Integrated PV–Battery Systems	Laminated flexible stack	Stable SOC with matched duty cycle	Continuous self-charging	Requires load optimization	Pulse oximeters, e-textiles	[79]

## Data Availability

No new data were created or analyzed in this study.
